# Synaptic lipids in cortical function and psychiatric disorders

**DOI:** 10.15252/emmm.201505749

**Published:** 2015-12-15

**Authors:** Bernardo Stutz, Tamas L Horvath

**Affiliations:** ^1^ Program in Integrative Cell Signaling and Neurobiology of MetabolismSection of Comparative MedicineYale University School of MedicineNew HavenCTUSA

**Keywords:** Genetics, Gene Therapy & Genetic Disease, Neuroscience

## Abstract

Psychiatric disorders, which include a variety of distinct infirmities, affect millions of people worldwide. Without intervention, the impact of these conditions is devastating, compromising the daily life of patients and their relatives. Although insights into the underlying cortical circuitry of psychiatric diseases have emerged over the years, our understanding of their pathophysiology, elucidation of pathophysiologic mechanisms and relevant advancements in clinical therapeutic strategies have been hampered by the complexity of these neural networks and the lack of reliable biomarkers in human subjects and animal models. In this issue of *EMBO Molecular Medicine*, Vogt and colleagues add significant new insights to the understanding of the etiology of psychiatric conditions by revealing novel contribution of synaptic lipids to altered circuit function and behavior in mice (Vogt *et al*, 2016).

Altered bioactive lipid metabolism and synaptic excitation/inhibition (E/I) imbalance in cortical areas have been associated with psychiatric disorders of patients and described in mouse models of psychiatric disorders (Calfa *et al*, 2015; Smesny *et al*, 2015). Proper E/I balance control is mandatory for normal cortical function; alterations in this balance can cause circuit dysfunction and disease. However, E/I ratio is dynamic rather than static, being fine‐tuned by mechanisms of synaptic plasticity. Bioactive lipids are thought to play a role in the regulation of synaptic plasticity and E/I balance control. Of particular interest is lysophosphatidic acid (LPA), a ubiquitous brain phospholipid involved in the regulation of synaptic plasticity, neural stem cell survival, neurite outgrowth and axonal guidance. LPA is produced by autotaxin and acts on a diverse set of receptors. Importantly, it is metabolized by post‐synaptic neurons through the activity of a member of the lipid phosphate phosphatase‐related proteins (LPPRs), also referred as plasticity‐related genes (PRGs).

PRG‐1 (LPPR‐4; EC:3.1.3.4) was the first identified member (Brauer *et al*, 2003) of the LPPR superfamily and so far is the only one known to possess ecto‐phosphatase activity (for review, see Strauss & Brauer, 2013). Its expression emerges perinatally, exclusively in the postsynaptic density of glutamatergic neurons in many brain areas, such as the hippocampus and entorhinal cortex (Brauer *et al*, 2003; Trimbuch *et al*, 2009) but also in the cerebellum and the neocortex (Tokumitsu *et al*, 2010). In addition to a phosphatase activity, a receptor/transporter activity has also been described for PRG‐1, suggesting an important role in the regulation of LPA‐mediated signaling and uptake, indicating a direct impact on excitatory inputs (Brauer *et al*, 2003; Trimbuch *et al*, 2009), development of anxiety‐like symptoms (Yamada *et al*, 2015) and psychiatric disorders, such as schizophrenia (Mirendil *et al*, 2015). In fact, many neurological disorders, including Alzheimer's disease, Parkinson's disease, depression, bipolar disorder, and schizophrenia, are characterized by altered phospholipid metabolism, accumulation of lipid peroxides, and increased levels of lysophospholipids, as well as hyperexcitation (increased E/I balance), excitotoxicity, oxidative stress, and inflammatory reactions (for review, see Ong *et al*, 2015).

In this issue of *EMBO Molecular Medicine*, Vogt and colleagues report a loss‐of‐function of the PRG‐1 enzyme caused by a single nucleotide polymorphism (SNP), which results in an arginine to threonine exchange at residue 345 (Vogt *et al*, 2016). This SNP affects around 5 million people in Europe and 1.5 million in the United States and it appears to be linked to impairments in sensory gating in human monoallelic carriers. Expressing the mouse homolog mutation of PRG‐1 (PRG‐1^R346T^), the authors observed compromised protein glycosylation and reduced enzymatic activity (Fig 1). Heterozygous PRG‐1‐deficient mice, the mouse correlate of human monoallelic PRG‐1^R345T^, display hyperexcitability in layer IV somatosensory barrel field mouse cortical neurons (altered E/I balance), as well as impaired sensory gating and social interaction. Because PRG‐1 regulates LPA levels and LPA has been demonstrated to cause symptoms related to neurological/psychiatric diseases, the authors used *in vitro* and *in vivo* inhibitors of LPA‐synthesizing enzyme autotaxin to decrease extracellular LPA levels and prevent biochemical, electrophysiological and behavioral alterations caused by R346T mutation/monoallelic depletion of the PRG‐1 gene in mice. These results shed critical new light on the importance of controlling synaptic LPA levels as well as the role of this phospholipid on mechanisms of plasticity and development of pathological states, which may affect the design of novel clinical interventions for psychiatric diseases in the near future.

**Figure 1:**
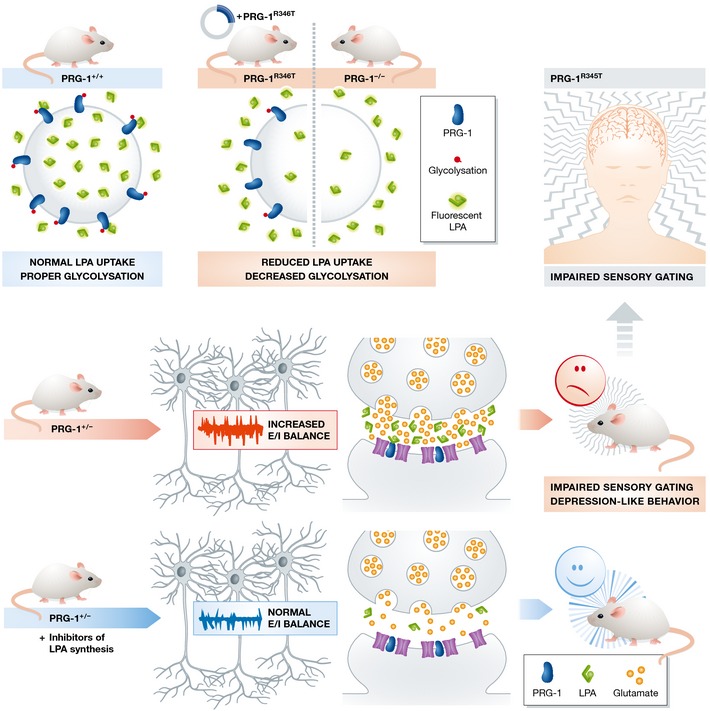
Lipid control of synaptic machinery. PRG‐1^R346T^: loss‐of‐function mutation leads to reduced LPA internalization due to altered PRG‐1 protein glycosylation. Mice heterozygous for PRG‐1 (mouse correlates of human PRG‐1^R345T^ loss‐of‐function mutation) have increased electrophysiological excitation/inhibition balance in somatosensory barrel field cortex neurons, display altered sensory gating, and endophenotypes typical for psychiatric disorders. All of these phenotypes were reversed by LPA synthesis (autotaxin) inhibitors. Humans carrying monoallelic PRG‐1^R345T^ mutation also display impaired sensory gating, suggesting a link between LPA levels and development of psychiatric disorders.

Most psychiatric disorders are moderately to highly heritable, indicating a strong genetic component along with a relevant gene–environment interplay in a significant fraction of disease development (for review, see Klengel & Binder, 2015). Adverse events, especially early in life, have consistently been shown to strongly increase the risk for mood and anxiety disorders in large epidemiological studies. Common to many early adversities are changes in long‐term regulation of stress hormones and changes in eating habits. Representing 60–70% of human brain dry weight, lipids are notably important for proper brain function. Circulating lipid species are modulated as a result of dietary habits. Because adipocytes are the major producers of circulating LPA, scenarios promoting adipocyte hypertrophy, such as high fat diet feeding, are thought to increase LPA levels. Circulating LPA may cause glucose intolerance, disrupt insulin secretion, suppress brown adipocyte differentiation, and promote diet‐induced obesity and diabetes (for review, see Rancoule *et al*, 2014). Inversely, LPA receptor KO mice fed a high fat diet (HFD) show no significant increase in body weight or fat mass and do not exhibit overconsumption of food associated with HFD feeding (Dusaulcy *et al*, 2009). Interestingly, diabetic obese human subjects display upregulation of autotaxin and increased levels of LPA, suggesting a positive feedback loop contributing to aggravate metabolic syndrome. Altogether, accumulating evidence suggests a crucial role for LPA, which can be modulated by diet, in the pathogenesis of central and peripheral diseases. Poor dietary habits are a risk factor for development of degenerative diseases, such as Alzheimer's disease, Parkinson's disease, and some forms of dementia (Kamel *et al*, 2014; Morris & Tangney, 2014). As such, healthy dietary habits and monitoring LPA levels could be potentially exploited for improved prevention, detection, and clinical treatment of psychiatric diseases.

## References

[emmm201505749-bib-0001] Brauer AU , Savaskan NE , Kuhn H , Prehn S , Ninnemann O , Nitsch R (2003) A new phospholipid phosphatase, PRG‐1, is involved in axon growth and regenerative sprouting. Nat Neurosci 6: 572–578 12730698 10.1038/nn1052

[emmm201505749-bib-0002] Calfa G , Li W , Rutherford JM , Pozzo‐Miller L (2015) Excitation/inhibition imbalance and impaired synaptic inhibition in hippocampal area CA3 of Mecp2 knockout mice. Hippocampus 25: 159–168 25209930 10.1002/hipo.22360 PMC4300269

[emmm201505749-bib-0003] Dusaulcy R , Daviaud D , Pradere JP , Gres S , Valet P , Saulnier‐Blache JS (2009) Altered food consumption in mice lacking lysophosphatidic acid receptor‐1. J Physiol Biochem 65: 345–350 20358347 10.1007/BF03185929

[emmm201505749-bib-0004] Kamel F , Goldman SM , Umbach DM , Chen H , Richardson G , Barber MR , Meng C , Marras C , Korell M , Kasten M *et al* (2014) Dietary fat intake, pesticide use, and Parkinson's disease. Parkinsonism Relat Disord 20: 82–87 24120951 10.1016/j.parkreldis.2013.09.023 PMC3936597

[emmm201505749-bib-0005] Klengel T , Binder EB (2015) Epigenetics of stress‐related psychiatric disorders and gene × environment interactions. Neuron 86: 1343–1357 26087162 10.1016/j.neuron.2015.05.036

[emmm201505749-bib-0006] Mirendil H , Thomas EA , De Loera C , Okada K , Inomata Y , Chun J (2015) LPA signaling initiates schizophrenia‐like brain and behavioral changes in a mouse model of prenatal brain hemorrhage. Transl Psychiatry 5: e541 25849980 10.1038/tp.2015.33 PMC4462599

[emmm201505749-bib-0007] Morris MC , Tangney CC (2014) Dietary fat composition and dementia risk. Neurobiol Aging 35(Suppl. 2): S59–S64 24970568 10.1016/j.neurobiolaging.2014.03.038 PMC4107296

[emmm201505749-bib-0008] Ong WY , Farooqui T , Kokotos G , Farooqui AA (2015) Synthetic and natural inhibitors of phospholipases A2: their importance for understanding and treatment of neurological disorders. ACS Chem Neurosci 6: 814–831 25891385 10.1021/acschemneuro.5b00073

[emmm201505749-bib-0009] Rancoule C , Dusaulcy R , Treguer K , Gres S , Attane C , Saulnier‐Blache JS (2014) Involvement of autotaxin/lysophosphatidic acid signaling in obesity and impaired glucose homeostasis. Biochimie 96: 140–143 23639740 10.1016/j.biochi.2013.04.010

[emmm201505749-bib-0010] Smesny S , Gussew A , Biesel NJ , Schack S , Walther M , Rzanny R , Milleit B , Gaser C , Sobanski T , Schultz CC *et al* (2015) Glutamatergic dysfunction linked to energy and membrane lipid metabolism in frontal and anterior cingulate cortices of never treated first‐episode schizophrenia patients. Schizophr Res 168: 322–329 26255566 10.1016/j.schres.2015.07.013

[emmm201505749-bib-0011] Strauss U , Brauer AU (2013) Current views on regulation and function of plasticity‐related genes (PRGs/LPPRs) in the brain. Biochim Biophys Acta 1831: 133–138 23388400 10.1016/j.bbalip.2012.08.010

[emmm201505749-bib-0012] Tokumitsu H , Hatano N , Tsuchiya M , Yurimoto S , Fujimoto T , Ohara N , Kobayashi R , Sakagami H (2010) Identification and characterization of PRG‐1 as a neuronal calmodulin‐binding protein. Biochem J 431: 81–91 20653564 10.1042/BJ20100637

[emmm201505749-bib-0013] Trimbuch T , Beed P , Vogt J , Schuchmann S , Maier N , Kintscher M , Breustedt J , Schuelke M , Streu N , Kieselmann O *et al* (2009) Synaptic PRG‐1 modulates excitatory transmission via lipid phosphate‐mediated signaling. Cell 138: 1222–1235 19766573 10.1016/j.cell.2009.06.050 PMC3716297

[emmm201505749-bib-0014] Vogt J , Yang JW , Mobascher A , Cheng J , Li Y , Liu X , Baumgart J , Thalman C , Kirischuk S , Unichenko P *et al* (2016) Molecular cause and functional impact of altered synaptic lipid signaling due to a *prg‐1* gene SNP. EMBO Mol Med 8: 25–38 26671989 10.15252/emmm.201505677 PMC4718157

[emmm201505749-bib-0015] Yamada M , Tsukagoshi M , Hashimoto T , Oka J , Saitoh A , Yamada M (2015) Lysophosphatidic acid induces anxiety‐like behavior via its receptors in mice. J Neural Transm 122: 487–494 25119538 10.1007/s00702-014-1289-9

